# The neurogenomic transition from territory establishment to parenting in a territorial female songbird

**DOI:** 10.1186/s12864-019-6202-3

**Published:** 2019-11-07

**Authors:** Alexandra B. Bentz, Douglas B. Rusch, Aaron Buechlein, Kimberly A. Rosvall

**Affiliations:** 1 Department of Biology, Indiana University, Bloomington, IN 47405 USA; 2 Center for the Integrative Study of Animal Behavior, Indiana University, Bloomington, IN 47405 USA; 3 Center for Genomics and Bioinformatics, Indiana University, Bloomington, Indiana USA

**Keywords:** Neural plasticity, Breeding stage, Competition, Incubation, SRD5A1, AVPR1A, CRH

## Abstract

**Background:**

The brain plays a critical role in upstream regulation of processes central to mating effort, parental effort, and self-maintenance. For seasonally breeding animals, the brain is likely mediating trade-offs among these processes within a short breeding season, yet research thus far has only explored neurogenomic changes from non-breeding to breeding states or select pathways (e.g., steroids) in male and/or lab-reared animals. Here, we use RNA-seq to explore neural plasticity in three behaviorally relevant neural tissues (ventromedial telencephalon [VmT], hypothalamus [HYPO], and hindbrain [HB]), comparing free-living female tree swallows (*Tachycineta bicolor*) as they shift from territory establishment to incubation. We additionally highlight changes in aggression-related genes to explore the potential for a neurogenomic shift in the mechanisms regulating aggression, a critical behavior both in establishing and maintaining a territory and in defense of offspring.

**Results:**

HB had few differentially expressed genes, but VmT and HYPO had hundreds. In particular, VmT had higher expression of genes related to neuroplasticity and processes beneficial for competition during territory establishment, but down-regulated immune processes. HYPO showed signs of high neuroplasticity during incubation, and a decreased potential for glucocorticoid signaling. Expression of aggression-related genes also shifted from steroidal to non-steroidal pathways across the breeding season.

**Conclusions:**

These patterns suggest trade-offs between enhanced activity and immunity in the VmT and between stress responsiveness and parental care in the HYPO, along with a potential shift in the mechanisms regulating aggression. Collectively, these data highlight important gene regulatory pathways that may underlie behavioral plasticity in females.

## Background

For seasonally breeding animals, rapidly shifting social and physical demands may lead to especially critical physiological, behavioral, and life history trade-offs [1, 2]. The breeding season often begins with intense social instability as animals compete for territories and mates [3, 4], and animals later shift to parenting while also maintaining their own energy reserves [5]. The central nervous system plays a critical role in upstream regulation of these processes [6, 7]; therefore, neural plasticity likely facilitates phenotypic adjustments across the breeding season as stage-specific selection pressures change. Indeed, some of the most pronounced examples of seasonal changes in the brain can be seen in male songbirds. A well-known example is the dramatic change in brain morphology and gene expression that occurs to promote singing behavior as the breeding season begins [8, 9]. The neurogenomic shift from non-breeding to breeding states has been relatively well-studied using both targeted gene approaches [10–12] and genome-wide studies [13, 14], but the shift from early breeding stages to later parental stages has received far less attention [15]. Moreover, past work examining changes within the breeding season, as males adjust to parental demands, has primarily focused on steroidogenic genes [16–18]. There are likely additional genes that play a critical, but currently unknown, role in the transition from a competitive to a parental brain.

It is likewise unclear how females mediate the shift from territory establishment to parenting, despite the fact that females provide the bulk of parental care in many systems. Seasonal work on females has been limited due to the relative lack of research on early breeding season competition [19] and the misconception that females are too “complex” given their reproductive endocrinology [20]. Yet this complexity is precisely why it is important to understand how the female brain changes from territory establishment to parenting. Researchers have begun to acknowledge that females also compete early in the breeding season to acquire breeding territories and maintain monogamy [21, 22], but, unlike most males, they have additional parental burdens that are energetically costly (e.g., offspring production and early care) [23, 24], generating potentially steep trade-offs. Much of our knowledge about female neural plasticity comes from work on captive animals transitioning to maternal roles. For example, poultry have been used to explore a role for dopamine- and prolactin-related genes in the onset of broodiness [25–28], and work in lab-reared female mammals suggests numerous hormones, neuropeptides, and neurotransmitters help “rewire” the maternal brain in preparation for offspring [29–31]. While there is some work in wild birds that demonstrates a genomic link between steroid-related genes and early spring behaviors (e.g., competitive aggression [32, 33]), genomic profiles during early breeding stages are relatively unknown. Thus, it remains to be tested what neurogenomic mechanisms track the transition from early breeding season competition to the steep parental demands that follow in a female experiencing a dynamic social environment.

Here, we identify neural genes and processes that may contribute to the transition from early territory establishment to parental care in females. We first compared genome-wide gene expression in brain tissues collected from free-living female tree swallows (*Tachycineta bicolor*) between a period when females were establishing territories but not yet fertile, and the incubation period. We focused on three brain regions – the hypothalamus (HYPO), ventromedial telencephalon (VmT), and hindbrain (HB) – that have been previously implicated in both early spring competition and later parental care behaviors [29, 34–36]. As a secondary goal, we examined expression of candidate genes related to aggression, as a window into how neurogenomic shifts may facilitate aggression across the breeding season. Aggression is a critical behavior both in establishing and maintaining a territory and in defense of offspring; yet, it is most often studied in relation to steroid hormones, like testosterone (T), which decline in circulation during the transition from territory establishment to parenting [37]. Comparisons between breeding and nonbreeding birds implicate neurosteroid-mediated pathways [10, 38–41], but numerous other processes, ranging from hormone signaling to energy metabolism, may also contribute to aggression [14, 35, 42–45]. Female tree swallows are especially amenable for addressing these knowledge gaps because they fiercely compete for limited nesting cavities early in the breeding season [46], prior to producing a single brood that females alone incubate [47]. Past experimental work in this system demonstrates aggression is mediated by T to some degree [48], but females can maintain moderately high levels of aggression during periods of low T, suggesting other mechanisms may play a role during maternal stages [49–52]. Thus, this system allows us to explore plasticity in the neurogenomic mechanisms that may facilitate the transition from territory establishment to parental care, as females experience a full suite of natural selection pressures.

## Results

### Gene expression patterns across breeding stages

**Fig. 1:**
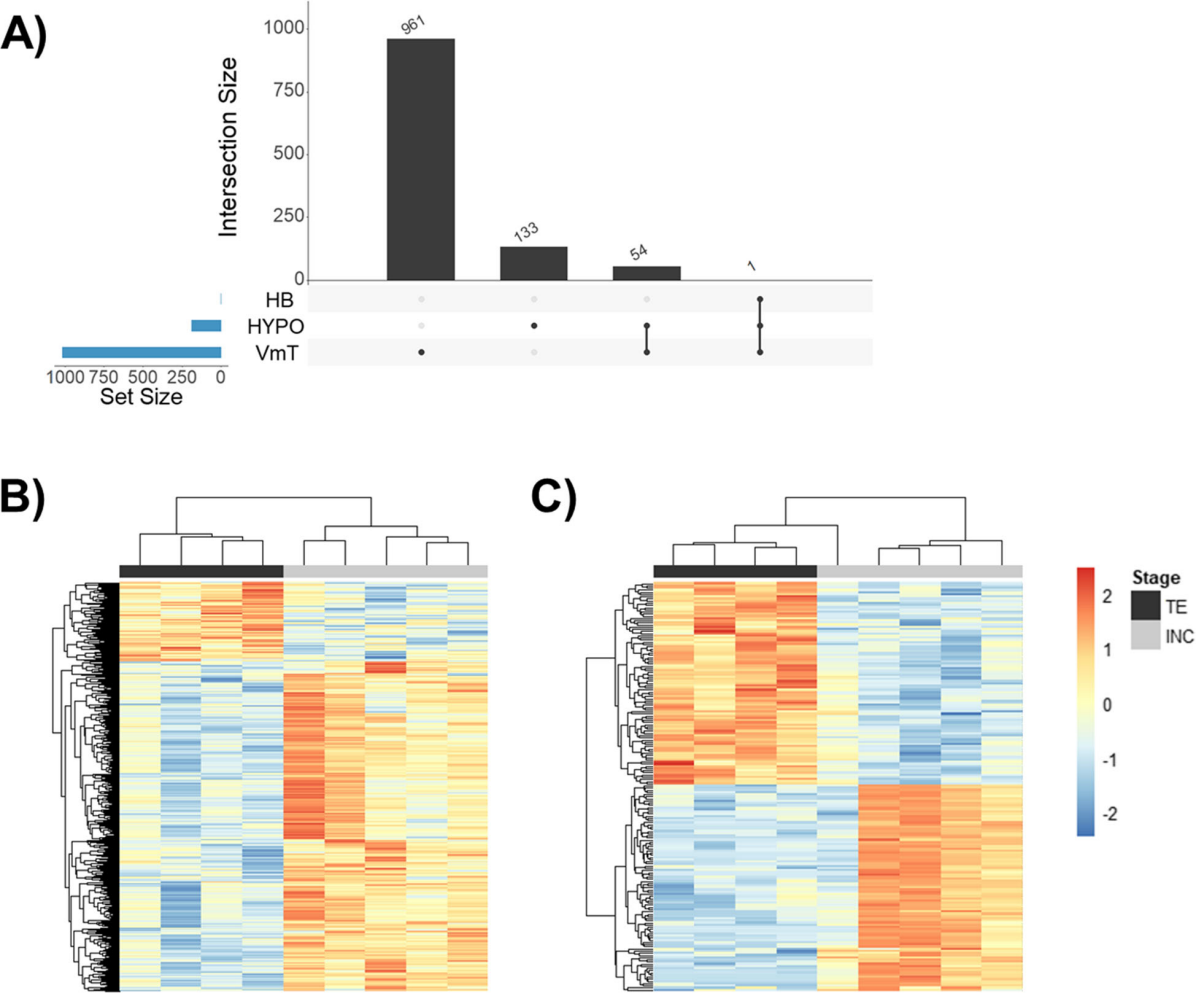
**a** UpSet plot depicting the number of unique and shared differentially expressed genes in the ventromedial telencephalon (VmT), hypothalamus (HYPO), and hindbrain (HB) from females collected during territory establishment (TE) and incubation (INC) breeding stages. Intersection size is the number of differentially expressed genes and the black dots on the x-axis represent whether these genes are present or absent in that set (i.e., VmT, HYPO, and/or HB). Heatmaps depicting differentially expressed genes between TE (dark gray) and INC (light gray) in (**b**) VmT and (**c**) HYPO. Each column is an individual and each row is a gene. Heatmaps are scaled across rows to allow for comparisons of gene expression across individuals. Color indicates log(FPKM+ 1); blue (lower) and red (higher) expression. Rows and columns are clustered using Euclidean distance

**Fig. 2:**
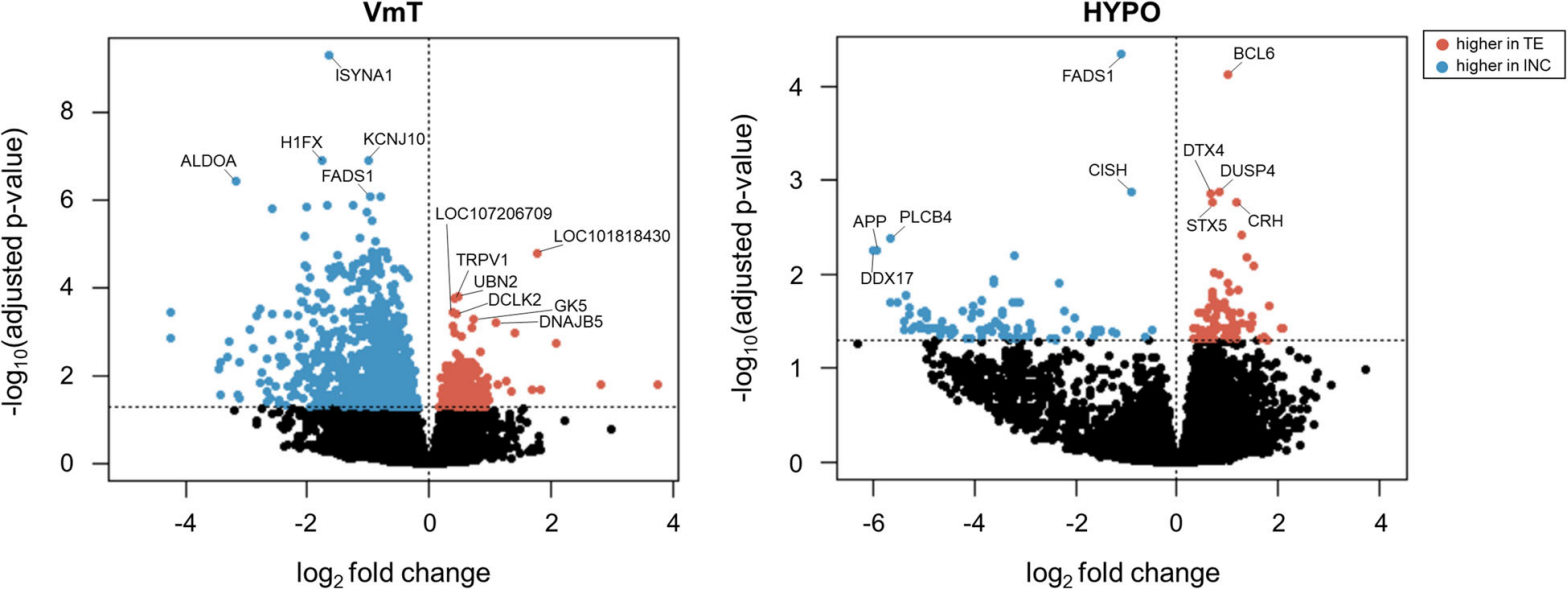
Volcano plots depicting the log_2_ fold change and corresponding -log_10_ adjusted *p*-value of all genes detected in the ventromedial telencephalon (VmT) and hypothalamus (HYPO). Differentially expressed genes (DEG; p_adj_ < 0.05) are shown in color (red, up-regulated in territory establishment [TE]; blue, up-regulated in incubation [INC]). Genes that did not have significant differences in expression between TE and INC are shown in black. The top 5 up- or down-regulated DEGs are annotated, using the LOC prefix for uncharacterized genes

Approx. 23 million read pairs per sample were mapped to the reference tree swallow transcriptome [53]. The VmT and HYPO showed the highest number of differentially expressed genes (DEG) between breeding stages, whereas the HB had only one DEG. The VmT had 1016 DEG (*n* = 196 genes up-regulated during territory establishment and *n* = 820 genes up-regulated during incubation; Fig. 1a, b). The most significant up-regulated genes during territory establishment in the VmT included genes involved in brain function (e.g., doublecortin like kinase 2; DCLK2), while those up-regulated during incubation included several genes involved in metabolism, like inositol-3-phosphate synthase 1 (ISYNA1), fructose-bisphosphate aldolase A (ALDOA), and fatty acid desaturase 1 (FADS1) (Fig. 2). The HYPO had 188 DEG (*n* = 93 genes up-regulated during territory establishment and *n* = 95 genes up-regulated during incubation; Fig. 1a, c). The most significant up-regulated genes during territory establishment in the HYPO were involved in immune function (e.g., B-cell lymphoma 6 protein; BCL6) and glucocorticoid signaling (e.g., corticotropin-releasing hormone; CRH), while those up-regulated during incubation were involved in synaptic plasticity, most notably amyloid beta precursor protein (APP) (Fig. 2). The VmT and HYPO shared 55 DEG (Fig. 1a), and the log_2_ fold change of these overlapping genes was significantly negatively related (linear regression: β = − 0.24, F_1,53_ = 4.04, *p* = 0.049). The single DEG in the HB (i.e., FADS1) was differentially expressed in all three brain regions and was consistently up-regulated during incubation (Fig. 1a). Due to there being only one DEG in the HB, subsequent analyses focus solely on the VmT and HYPO. For the full list of DEG, see Additional file 1: Table S1.

### Functional enrichment analyses of DEGs

**Fig. 3:**
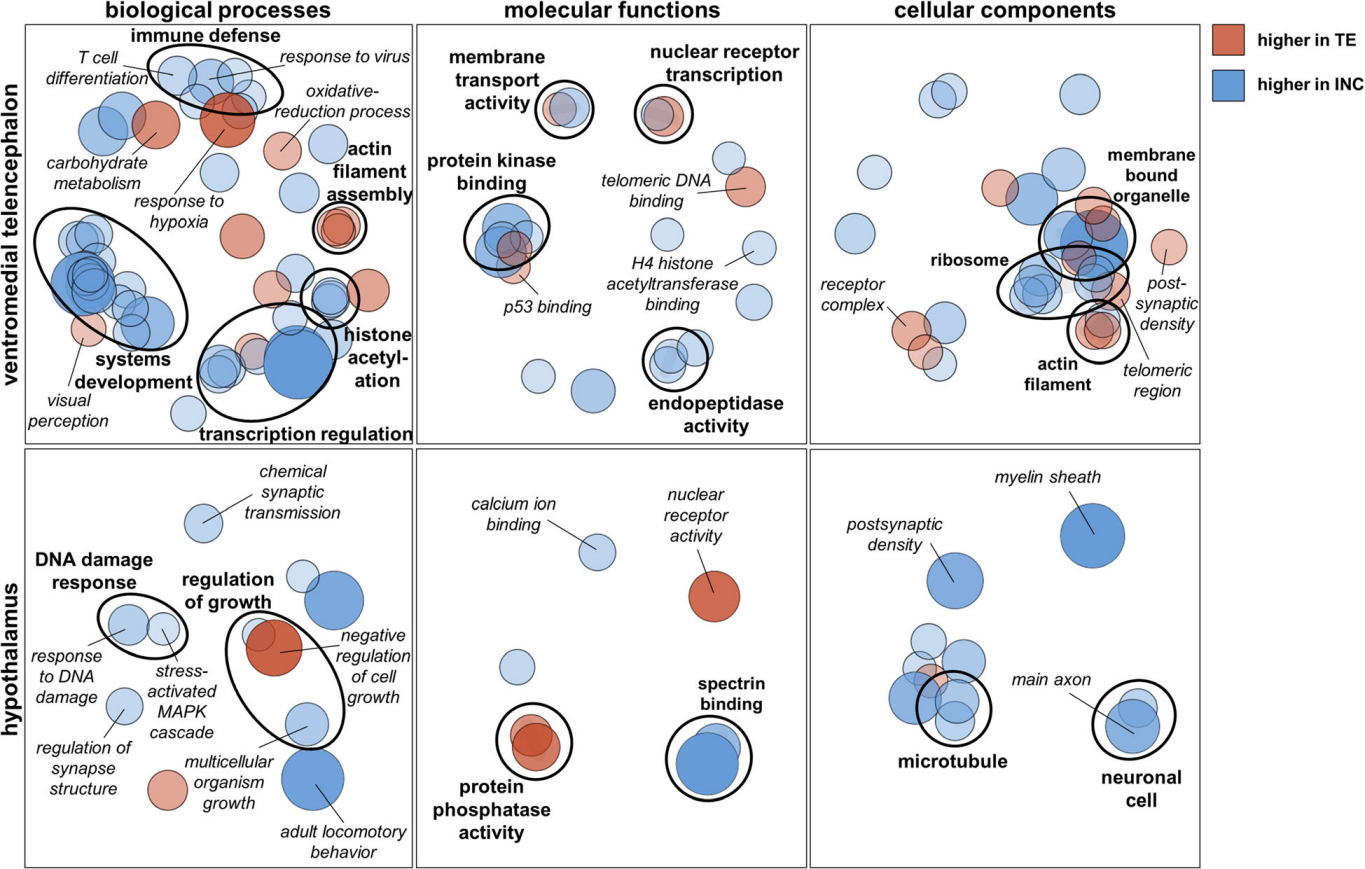
Major biological processes, molecular functions, and cellular components regulated in response to breeding stage (territory establishment [TE] or incubation [INC]) for each brain region as revealed by Gene Ontology (GO) analyses. Each circle represents a GO term; color and shading indicate significance (larger and darker is more significant, all *p* < 0.01). GO terms are clustered based on semantic similarity. All GO terms have > 3 genes. See Additional file 1: Table S2 for the full list

In the VmT and HYPO, where hundreds of genes were differentially expressed between breeding stages, we analyzed biological process, molecular function, and cellular component Gene Ontology (GO) terms for all genes with differential patterns of expression between territory establishment and incubation. In the VmT, DEGs up-regulated during territory establishment were enriched for terms related to activity (e.g., visual perception, response to hypoxia, and carbohydrate metabolic processes), neuroplasticity (e.g., actin filament and postsynaptic density), and cellular damage (e.g., telomeric DNA binding and oxidation-reduction processes) (Fig. 3). DEG up-regulated during incubation in the VmT contributed to processes like immune defense (e.g., CD4-positive T cell differentiation and response to virus) and transcriptional regulation, including histone acetylation (Fig. 3). In the HYPO, DEG related to nuclear receptor activity and negative regulation of growth were highly expressed during territory establishment, while DEG related to neuroplasticity (e.g., myelin sheath, microtubules, and postsynaptic density), signaling (e.g., chemical synaptic transmission and stress-activated MAPK signaling), and DNA damage were highly expressed during incubation (Fig. 3). For a full list of GO terms, see Additional file 1: Table S2.

### Candidate genes in the context of aggression

**Fig. 4:**
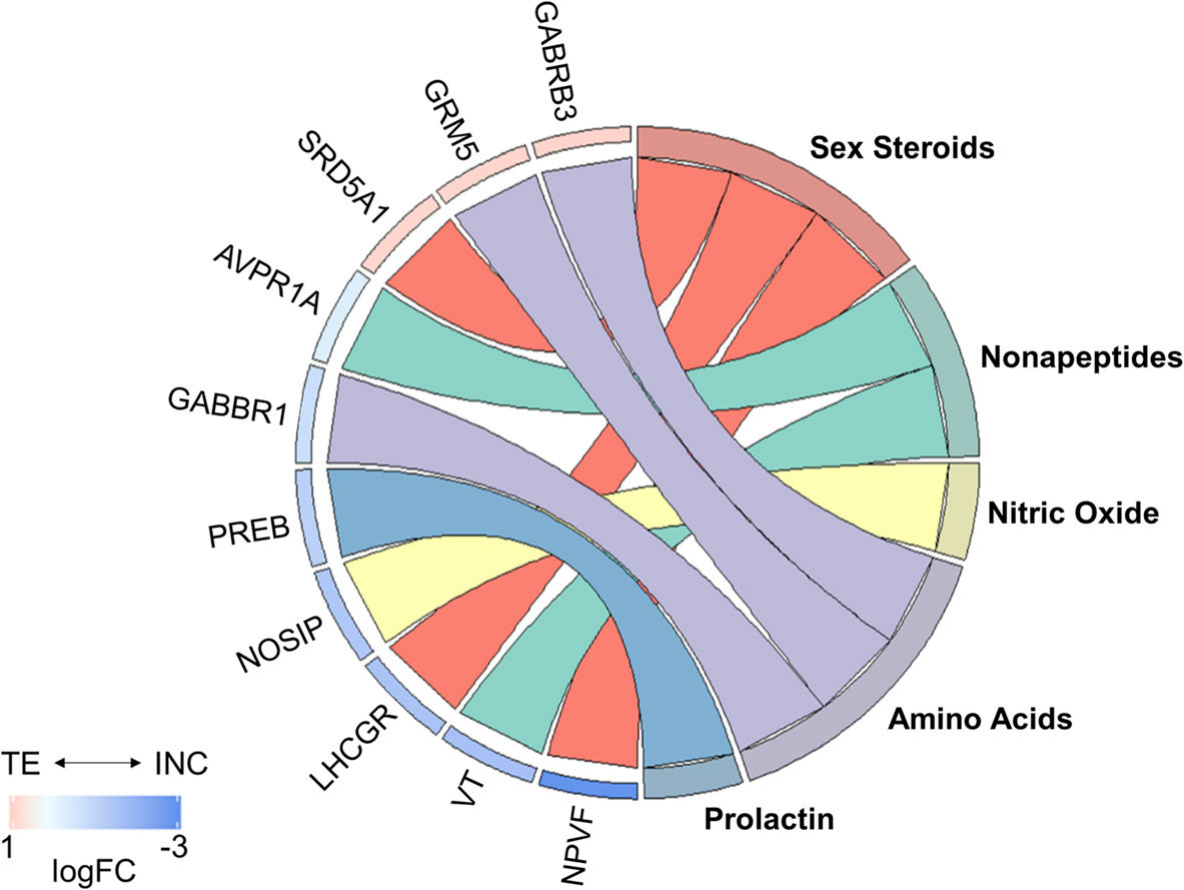
GOChord plot of candidate aggression genes differentially expressed in the ventromedial telencephalon (all p_adj_ < 0.05). The genes are linked to their assigned pathway via colored ribbons. Genes are ordered according to the observed log_2_ fold change (LogFC), which is displayed in descending intensity next to the selected genes from red (higher expression during territory establishment [TE]) to blue (higher expression during incubation [INC])

We manually investigated the list of DEG for *a priori* identified candidate genes that had previously been implicated in the neurobiological control of aggression, including genes along the following pathways: catecholamines, serotonin, amino acids, nitric oxide, sex steroids, glucocorticoids, nonapeptides, histamine, neurotrophic factors, prolactin, and opioids [36]. We identified 81 genes along these pathways in our dataset (see Additional file 1: Table S3). Within the VmT, key sex steroid and glutamate signaling genes were up-regulated during territory establishment (5α-reductase [SRD5A1] and glutamate metabotropic receptor 5 [GRM5]); however, we saw up-regulation of genes in non-steroidal pathways during incubation, including prolactin-related signaling (prolactin regulatory element binding [PREB]) and nonapeptide-related signaling (i.e., vasotocin [VT] and arginine vasopressin receptor 1A [AVPR1A]), although the fold change for AVPR1A was low (Fig. 4). The only differentially expressed candidate aggression-related gene in the HYPO was related to glucocorticoid signaling (i.e., CRH), which was up-regulated during territory establishment (Fig. 2).

## Discussion

The adult vertebrate brain is capable of extensive seasonal neuroplasticity, yet little is known about the underlying genomic changes that occur within the breeding season, particularly in females. Here, we show that expression of hundreds of genes in VmT and HYPO, but not HB, change as females shift from competing for territories to providing parental care. Many of these genes are associated with neuroplasticity-related processes highlighted in previous research on the transition from non-breeding to breeding condition in male songbirds [13, 14], suggesting these pathways may be relevant even on relatively short timescales. We additionally found genomic patterns suggestive of potentially important trade-offs between metabolism/activity and self-maintenance. Finally, our results show candidate genes related to aggression are more diverse later in the breeding season, suggesting a switch to non-steroidal mechanisms of aggression, similar to what is seen in the mammalian maternal brain [36]. Together, these data offer insight into the diverse neurogenomic mechanisms that accommodate the shift from competitor to parent in females, which endure the strongest reproductive costs but are paradoxically the least studied of the sexes.

The brain region that showed the greatest plasticity in relation to breeding stage was the VmT. This region is strongly associated with the regulation of social behaviors [34, 54], and past work in female birds shows high immediate early gene activity in this region in response to conspecific intrusions [55]. We found that many more genes were up-regulated during incubation compared to territory establishment. This genomic plasticity may have been facilitated by epigenetic mechanisms, as genes associated with histone acetylation, which typically make genes more accessible to transcription [56], were among those up-regulated during incubation. One explanation for why there would be more up-regulated genes during incubation could be that females are shifting to a more “complex” maternal brain, with enhanced cognitive flexibility, emotional regulation, and social attentiveness [57]. Alternatively, regulation of social behavior may occur through a more refined pathway requiring fewer neurogenomic changes during territory establishment, allowing females to quickly respond to intruders. This idea is consistent with our finding that fewer candidate genes for aggression are up-regulated during territory establishment. Likewise, this echoes a report on male song sparrows (*Melospiza melodia*), which responded to territorial challenges with fewer DEG during the breeding season than the non-breeding season [14]. Regardless, our data highlight the potentially important role of neurogenomic plasticity in the VmT in females.

The brain plays a critical role in upstream regulation of many of the processes central to competition, parental care, and self-maintenance, and likely facilitates phenotypic adjustments in these traits across the breeding season. We found that during territory establishment, when females are actively engaged in acrobatic aerial fights, genes related to neuroplasticity (e.g., actin filament and postsynaptic density) and glutamate receptors were up-regulated in the VmT, suggestive of high neural activity. We also found that DCLK2, a member of a family of doublecortin genes commonly used as a proxy of neurogenesis [58, 59], had higher expression during territory establishment. Enhanced neuroplasticity in the VmT during a time of social instability could reflect a high demand for processing social information [60–62]. In support of this, we found high expression both of a DnaJ heat shock protein (DNAJB5) previously shown to be elevated when animals observe fights [63] and of a glutamate receptor (GRM5) that may encode social experiences to promote stronger responses to future aggression [64, 65]. Additionally, DEG up-regulated during territory establishment were enriched for processes that could facilitate aggression, like carbohydrate metabolism, visual perception, and response to hypoxia. This is consistent with observations that central processing of visual and other sensory signals are important during social interactions [66], and metabolic processes are an evolutionarily conserved response to territory defense [45]. However, elevated activity and metabolism likely come at a cost. High neural activity can make neurons more susceptible to DNA damage and cell death [67]. Accordingly, functions related to cellular damage, like p53 binding and telomeric DNA binding, were enriched during territory establishment. Furthermore, genes related to immune processes (e.g., T cell differentiation and viral responses) were down-regulated during territory establishment relative to incubation, suggesting that self-maintenance may be deprioritized during territory establishment. Alternatively, females may be more immune-challenged during incubation causing immune processes to be up-regulated during this stage relative to territory establishment. In either case, these findings suggest females may favor more energetically expensive states in the VmT during territory establishment, and this may come at the expense of self-maintenance.

The HYPO also showed signs of potential trade-offs between physiology and behavior, particularly during incubation, when genes associated with neuroplasticity were elevated. The HYPO is an important regulator of reproductive and parental behaviors [34], which may explain why mechanisms of neural activity were increased during this stage. Many of the genes up-regulated during incubation were associated with neuronal components (e.g., postsynaptic density, microtubules, and myelin sheath) and processes (e.g., synaptic transmission and MAPK signaling), along with decreased expression of genes that negatively regulate cell growth. We also found indications of a cost to enhanced neural plasticity, as cellular DNA damage response was also enriched. These findings largely parallel those of seasonal comparisons between breeding and non-breeding male songbirds. For example, microtubules [14] and cell growth-related processes [13] have also been suggested to play a role in increased seasonal neuroplasticity and morphological changes in the song control center, respectively. Protein kinases, such as those involved in the MAPK signaling pathway found here, are also implicated in the control of synaptic plasticity in the adult brain [68]. Additionally, one of the most significant DEG during incubation was APP, which plays an important role in neuronal plasticity and may be involved in social responsiveness during the non-breeding season [14]. However, our data suggest that such plasticity may come at some cost. For instance, CRH, which activates the hypothalamic-pituitary-adrenal (HPA) axis, was down-regulated during incubation. Many seasonally breeding birds attenuate the stress response during reproduction because it tends to inhibit reproductive and parental functions [69], but this may also leave animals without appropriate coping mechanisms [70]. While stress responsiveness across the breeding season remains to be more directly tested in tree swallows, parental behaviors are negatively affected by brief elevations in corticosterone [71, 72]. Thus, females may benefit from suppressing the HPA axis during this time. Altogether, our data show that mechanisms of neuroplasticity are heighted in the HYPO during incubation, perhaps to shift trade-off resolution away from self-maintenance and toward parental care.

The HB showed the lowest plasticity across the breeding season, despite past work suggesting it is socially responsive [35]. The HB’s vital role in basic functioning, such as energy balance and various autonomic processes [73], may ensure a genomic equilibrium is generally maintained across breeding stages. We did find one DEG (i.e., FADS1), which had significantly higher expression during incubation in all three brain regions. FADS1 encodes a key enzyme in fatty-acid synthesis, which is essential for proper brain functioning. It is not yet clear whether this shift relates to the putatively higher cognitive demands of the maternal brain [57] or it may instead reflect seasonal dietary changes toward aquatic insects, which have higher levels of fatty acid precursors [74, 75]. The up-regulation of this gene during incubation across three distinct areas of the brain suggests that it is likely critical to the transition across the breeding season.

As a secondary goal, we examined expression of aggression-related genes across the breeding season. Female tree swallows are an ideal system for examining plasticity in mechanisms of aggression because they remain defensive of their territories throughout the breeding season [51], but have much lower circulating T levels during incubation compared to the early breeding season [49, 50]. Most research on the seasonal plasticity of aggression has focused on sex steroids [10, 16–18, 38–41]. We did find that SRD5A1, which converts T into the potent androgen 5α-dihydrotestosterone (DHT), was upregulated in the VmT during territory establishment, when T levels are relatively high [49, 50]. These data point to DHT as a fruitful avenue for future research on female aggression during territory establishment. However, we found no evidence of enhanced neural steroid processing during the incubation period. The steroid-related genes that were up-regulated during incubation are either known inhibitors of gonadotropins (neuropeptide VF precursor; NPVF) or have unknown functions in the brain (luteinizing hormone receptor; LHCGR [76]). This contrasts with comparisons of breeding and non-breeding songbirds, where neurosteroid-mediated functions likely play a role in aggression when systemic T is low [10, 38–41]. Instead, our data are consistent with a switch to non-steroidal mechanisms to facilitate aggression during parental periods. Evidence from mammalian studies suggests numerous pathways that play a role in maternal care behaviors can also be co-opted for maternal aggression [36]. In agreement with this, we found that key genes associated with vasotocin (AVPR1A and VT) and prolactin (PREB, activates the prolactin promoter [77]) were up-regulated during incubation. Vasopressin (homologous to avian vasotocin) is strongly associated with aggression during periods of parental care in mammals [78–80], and some studies have found a link between vasotocin and aggression in birds, although these mechanisms may be species- and context-specific [81–83]. Prolactin has also been associated with maternal aggression in mammals [36, 80, 84] and birds [85, 86]. While direct links between these genes and aggression during incubation remain to be tested, these data demonstrate that female songbirds display the potential to use mechanisms of aggression that are similar to those used by female mammals during parental periods.

## Conclusions

Using a free-living, territorial female songbird, we present a comprehensive analysis of gene expression in socially relevant brain regions and how it changes across distinct breeding stages. We found evidence of neuroplasticity on par with that seen in males as they transition from non-breeding to breeding states. In addition, specific genomic signatures reveal how the female brain may resolve trade-offs between territorial aggression, self-maintenance, and parenting. As one of the first genome-wide analyses of breeding stage neuroplasticity in free-living females, these data shed new light on the genomic mechanisms that may contribute to the remarkable physiological and behavioral plasticity that occur as females transition from territory establishment to parenting.

## Methods

### Sample collection

We used 10 free-living adult female tree swallows (mass = 19.13 g ± 0.47 SE) captured during territory establishment early in the breeding season (*n* = 5 females) and during incubation (*n* = 5). Females collected during territory establishment were actively engaging in aggressive interactions at nest boxes (KAR, pers. obs.) and had recrudesced ovaries with small white follicles. Females collected during incubation had clutches of 4–6 eggs completed 3–11 days earlier out of the typical 14-day incubation period. Females were trapped in their nest boxes located in Monroe and Brown County, Indiana (39°9 N, 86°31 W) in April and May 2016 between 0900 and 1200 h. Females were euthanized with an overdose of isoflurane, followed by decapitation. Tissues were immediately collected, and brains were frozen on powdered dry ice in the field and transferred to -80 °C in the lab. All samples were collected under the approval of the Federal Fish and Wildlife Migratory Bird Permit #MB59069B-0, Indiana Scientific Purposes License #16–104, and the Indiana Department of Natural Resources. All research activities were approved by the Bloomington Institutional Animal Care and Use Committee under protocol #15–004.

### Tissue dissection, RNA isolation, and sequencing

Brains were macrodissected into functional regions, following [12, 38]. After removing the optic tecta and optic chiasm, we collected the HB at the level of the mammillary bodies, and we isolated the HYPO to the depth of the anterior commissure (including the preoptic area and ventromedial HYPO). We also collected the VmT by removing 1 mm of the ventromedial portion of the caudal telencephalon, based on the position of nucleus taeniae in other songbirds [12]. We focused on these tissues because they include nuclei that are involved in the regulation of hormones, behaviors, and energy balance, and they have been shown to respond to social challenges in earlier work [34–36]. Total RNA was extracted from each sample separately using the phenol-chloroform-based Trizol method, following the manufacturer’s instructions (Invitrogen, Carlsbad, CA). Total RNA was resuspended in water, and quality and quantity of RNA were analyzed with an Agilent 2200 TapeStation (Agilent Technologies, Santa Clara, CA). Total RNA was submitted to Indiana University’s Center for Genomics and Bioinformatics for cDNA library construction using a TruSeq Stranded mRNA HT Sample Prep Kit (Illumina) following the standard manufacturing protocol. Sequencing was performed using an Illumina NextSeq 500/550 Kit v2 with a 75 bp sequencing module generating 38 bp paired-end reads. After the sequencing run, demultiplexing was performed with bcl2fastq v2.20.0.422. One sample collected during territory establishment had a RIN < 3.4 in the HB and HYPO (all other RIN > 9.0), and this sample deviated strongly from the other samples (Additional file 2: Figure S1); therefore, we removed this female (*n* = 4 territory establishment).

### RNA-seq mapping and differential gene expression

Removal of adapter sequences and quality trimming was performed on NextSeq read sequences using Trimmomatic version 0.36 [87]. The resulting reads were mapped to the reference tree swallow transcriptome obtained previously by our laboratory [53] using Bowtie2 version 2.3.4.1 [88]. Results were filtered to only included reads mapped in proper pairs, sorted, and indexed using Samtools version 1.3.1 [89]. Approx. 23 million read pairs per sample were mapped, which account for ~ 81% of the total trimmed read pairs (Additional file 1: Table S4). Custom perl scripts were used to count mapped reads to transcripts and estimate transcript abundances based on Fragments Per Kilobase of exon per Million fragments mapped (FPKM). Transcripts with less than 10 total reads across samples were filtered. Using the *DESeq2* package (version 1.16.1) in R/Bioconductor (R version 3.4.1), we fit a negative binomial generalized linear model with a fixed effect for tissue and breeding stage for each transcript and calculated per-transcript Wald test statistics to identify significant differences [90]. *P*-values were corrected using Benjamini-Hochberg corrections and genes with an adjusted *p*-value ≤0.05 were considered as statistically differentially expressed.

### DEG enrichment analyses

GO enrichment analyses (biological process, molecular function, and cellular component) were performed separately for up- and down-regulated DEGs in the VmT and HYPO using the *topGO* package in R with the weight01 algorithm and a Fisher’s exact test (cut-off of *p* < 0.01 [91];). We removed GO terms with < 3 genes. We used REVIGO to reduce redundancy between GO terms (similarity cut-off of 0.9) and to cluster them based on semantic similarity [92]. Clustered terms were visualized in Cytoscape (v3.7.0) and annotated with AutoAnnotate [93].

## Supplementary information

**Additional file 1: Table S1.** Differentially expressed genes between territory establishment and incubation in the ventromedial telencephalon(VmT),hypothalamus(HYPO), and hindbrain(HB). **Table S2.** Old change were analyzed . The most specific terms with significant effects(Fisher’s adjusted *p* < 0.001) and that contain > 3 genes are reported. Ont = Ontology (BP = Biological Process; CC = Cellular Component.**Table S3.** List of the candidate aggression-related genes detected in our dataset. The list of relevant pathways was obtained from the literature (Nelson,2005;Filby et al.,2010) and functions are from GeneCards (https://www.genecards.org)**Table S4.** RNA-Seq mapping statics, including total read pairs from the sequencer, total read pairs after trimming, total read pairs mapped(both reads in the pair mapped to the same manuscript),and percentage mapped.

**Additional file 2: Figure S1.** Correlation matrix between samples with (A) and without (B) the outlier female #85692. Heat maps display Spearman rank correlations between all pairwise comparisons for all tissues and breeding stages. Spearman correlations were calculated using the log_2_(normalized read counts) and the genes with the lowest 25% variance were removed.

## Data Availability

Raw sequence reads can be obtained from the Gene Expression Omnibus database (GEO accession number GSE134939). The datasets used in the current study are either available in its supplementary information files or will be made available from the corresponding author on reasonable request.

## Abbreviations

ACTN1: Alpha-actinin-1
ALDOA: Fructose-bisphosphate aldolase A
APP: Amyloid beta precursor protein
AVPR1A: Arginine vasopressin receptor 1A
BCL6: B-cell lymphoma 6 protein
CDC42BPA: Serine/threonine-protein kinase MRCK alpha
CISH: Cytokine-inducible SH2-containing protein
CRH: Corticotropin-releasing hormone
DCLK2: Doublecortin like kinase 2
DDX17: Probable ATP-dependent RNA helicase DDX17
DEG: Differentially expressed genes
DHT: 5α-dihydrotestosterone
DNAJB5: DnaJ heat shock protein
DTX4: E3 ubiquitin-protein ligase DTX4
DUSP4: Dual specificity protein phosphatase 4
EBP: 3-beta-hydroxysteroid-Delta-8,Delta-7-isomerase
FADS1: Fatty acid desaturase 1
FERRITIN H: Ferritin, higher subunit
FPKM: Fragments per kilobase of exon per million fragments mapped
GABBR1: Gamma-aminobutyric acid type B receptor subunit 1
GABRB3: Gamma-aminobutyric acid receptor subunit beta-3
GK5: Glycerol kinase 5
GO: Gene ontology
GRM5: Glutamate metabotropic receptor 5
H1FX: H1 Histone Family Member X
HB: Hindbrain
HPA: Hypothalamic-pituitary-adrenal
HYPO: Hypothalamus
INC: Incubation
ISYNA1: Inositol-3-phosphate synthase 1
KCNJ10: ATP-sensitive inward rectifier potassium channel 10
LHCGR: Luteinizing hormone receptor
NOSIP: Nitric oxide synthase-interacting protein
NPVF: Neuropeptide VF precursor
PLCB4: 1-phosphatidylinositol 4,5-bisphosphate phosphodiesterase beta-4
PREB: Prolactin regulatory element binding
RFT1: RFT1 homolog
RWDD2A: RWD domain-containing protein 2A
SRD5A1: 5α-reductase
STX5: Syntaxin-5
T: Testosterone
TE: Territory establishment
TRPV1: Capsaicin receptor
UBN2: Ubinuclein-2
VmT: Ventromedial telencephalon
VT: Vasotocin
VTN: Vitronectin
XRN1: 5′-3′ exoribonuclease 1
