# Supplementary material for: The neurogenomic transition from territory establishment to parenting in a territorial female songbird
Source: BMC Genomics. 2019 Nov 7;20:819. doi: 10.1186/s12864-019-6202-3 (PMC6836416; doi:10.1186/s12864-019-6202-3)
Supplement: Supplementary file 2 — Additional file 2: Figure S1. Correlation matrix between samples with (A) and without (B) the outlier female #85692. Heat maps display Spearman rank correlations between all pairwise comparisons for all tissues and breeding stages. Spearman correlations were calculated using the log2(normalized read counts) and the genes with the lowest 25% variance were removed. [file 12864_2019_6202_MOESM2_ESM.docx]

**Supplementary Materials**: The neurogenomic transition from territory establishment to parenting in a territorial female songbird

Alexandra B. Bentz^1,2*^, Douglas B. Rusch^1,3^, Aaron Buechlein^3^, and Kimberly A. Rosvall^1,2^

^1^Department of Biology, Indiana University, Bloomington, IN 47405, USA

^2^Center for the Integrative Study of Animal Behavior, Indiana University, Bloomington, IN 47405, USA

^3^Center for Genomics and Bioinformatics, Indiana University, Bloomington, Indiana, USA

Corresponding author^*^: bentza@iu.edu (ABB)


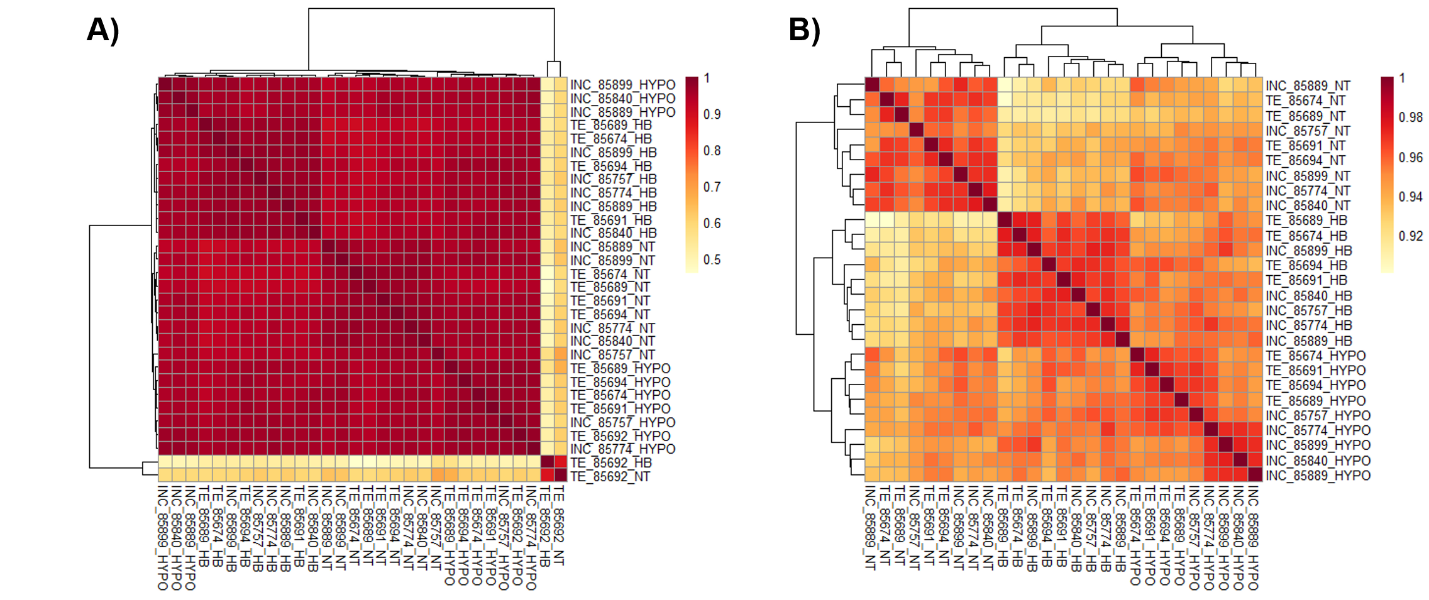


**Figure S1**. Correlation matrix between samples with (A) and without (B) the outlier female #85692. Heat maps display Spearman rank correlations between all pairwise comparisons for all tissues and breeding stages. Spearman correlations were calculated using the log_2_(normalized read counts) and the genes with the lowest 25% variance were removed.
